# Secondary B-cell lymphoma associated with the Epstein-Barr virus in chronic lymphocytic leukemia patients

**DOI:** 10.1007/s12308-016-0273-8

**Published:** 2016-05-21

**Authors:** Julie Morscio, Emilie Bittoun, Nathalie Volders, Eveline Lurquin, Iwona Wlodarska, Olivier Gheysens, Peter Vandenberghe, Gregor Verhoef, Philippe Demaerel, Daan Dierickx, Xavier Sagaert, Ann Janssens, Thomas Tousseyn

**Affiliations:** 1 Department of Imaging and Pathology, Lab for Translational Cell and Tissue Research, KU Leuven, Leuven, Belgium; 2 Department of Pathology, University Hospitals Leuven, Leuven, Belgium; 3 Center of Human Genetics, University Hospitals Leuven, Leuven, Belgium; 4 Department of Nuclear Medicine, University Hospitals Leuven, Leuven, Belgium; 5 Hematology Department, University Hospitals Leuven, Leuven, Belgium; 6 Department of Radiology, University Hospitals Leuven, Leuven, Belgium

**Keywords:** Epstein-Barr virus, Chronic lymphocytic leukemia, Richter transformation, Immunosuppression-related lymphoproliferation, Immunomodulatory agent-related lymphoproliferation, Immunodeficiency-related lymphoma

## Abstract

Up to 10 % of chronic lymphocytic leukemia (CLL) patients present with aggressive secondary B-cell lymphoma (most frequently diffuse large B-cell lymphoma, DLBCL) which may be clonally related to the CLL (i.e., Richter transformation, RT, 80 % of the cases) or de novo (20 % of the cases). Several genetic lesions associated with RT have already been identified, but the potential role of the Epstein-Barr virus (EBV) has been largely overlooked.

In this study, we describe six CLL patients who developed a secondary EBV-positive (EBV^+^) B-cell lymphoma (five DLBCL, one Hodgkin lymphoma) and compare their clinicopathological characteristics to ten CLL patients with EBV-negative (EBV^−^) secondary B-cell lymphomas (all DLBCL).

All 16 patients had a history of iatrogenic immunosuppression or chemotherapy. Eighty percent had received fludarabine as part of the CLL treatment. Most secondary lymphomas were clonally related to the previous CLL (3/4 EBV^+^, 7/7 EBV^−^ cases tested). Notably EBV^+^ RT was associated with a trend for older age at onset (median 72 vs. 63 years, *p* value >0.05), longer interval between CLL and RT diagnosis (median 4.2 vs. 2.9 years, *p* value >0.05), and shorter overall survival (median 4 vs. 10 months, *p* value >0.05). These differences were not significant, probably due to small sample size. Immunohistochemical profiling suggested more frequent overexpression of TP53 and MYC in EBV^−^ compared to EBV^+^ secondary lymphoma.

Based on this small retrospective single center series, we hypothesize that EBV^+^ RT may constitute a separate subgroup of RT. Larger series are required to validate this suggestion.

**Electronic supplementary material:**

The online version of this article (doi:10.1007/s12308-016-0273-8) contains supplementary material, which is available to authorized users.

## INTRODUCTION

Up to 10 % of patients with B-cell chronic lymphocytic leukemia (CLL), the most common type of adult leukemia, eventually develop an aggressive secondary lymphoma (most commonly diffuse large B-cell lymphoma, DLBCL) referred to as Richter transformation (RT) [1]. Although currently both clonally related and clonally unrelated secondary lymphomas are referred to as RT, recent (epi)genetic studies have demonstrated that these are actually distinct entities [2, 3]. This is also apparent from the clinical setting as “true,” i.e., clonally related cases of RT have a dismal prognosis compared to clonally unrelated DLBCL, which follows a disease course similar to de novo DLBCL [4].

In recent years, a number of risk factors for RT development have been defined including loss/mutation of *TP53* [5, 6], *NOTCH1* mutations [7], and the presence of the unmutated immunoglobulin heavy variable gene IGHV4–39, which is frequently part of the B-cell receptor in the DLBCL variant of RT [8]. These observations may be clinically useful for prompt recognition of RT as the outcome is influenced by the extent of disease [9, 10]. Regarding RT pathogenesis, several genetic lesions have been identified [11]. Notably, a few studies have addressed the potential role of the Epstein-Barr virus (EBV), an oncogenic human herpesvirus, in this process. However, because EBV is only occasionally detected in cases of RT [12, 13], its role as a driving factor is unclear. In vitro studies have shown that CLL cells are not easily infected by EBV and that cytokine signaling in the microenvironment may be crucial for rendering CLL cells susceptible to EBV infection [14]. Rossi et al. suggested that EBV-positive lymphoma in the context of CLL represents a novel type of immunodeficiency-related lymphoma that may develop following T-cell depletion or chemotherapy [9]. Following an intriguing case of EBV-related large B-cell transformation in the brain of a CLL patient, we reviewed all cases of secondary B-cell lymphoma in CLL patients available in our archive. This series was screened for the presence of EBV in an attempt to determine the clinicopathologic characteristics and possible prognostic implications of the involvement of EBV. Based on our results, we suggest that EBV-positive (EBV^+^) RT may represent a distinct subgroup of RT. We therefore encourage monitoring of EBV titers in newly diagnosed CLL and systematic screening for EBV in RT, particularly in elderly patients, to validate this hypothesis in a larger series of patients.

## Materials and methods

### Patient selection, histopathology, and statistics

In this retrospective single-center study, the clinicopathologic characteristics of 16 secondary B-cell lymphoma cases (retrieved from the archives of the University Hospitals Leuven) are described. All slides were reviewed by an expert hematopathologist (TT), and diagnosis was made according to the 2008 WHO criteria [15]. Clinical information was retrieved for all cases from the medical records. The Ethical Committee of the University Hospitals of Leuven approved this study (S-55498) which was conducted according to the Declaration of Helsinki.

EBER (EBV-encoded RNA) in situ hybridization was performed using a 30-mer digoxigenin-labeled oligonucleotide probe (Research Genetics, Huntsville, AL), according to the manufacturer’s instructions. To check the RNA integrity, a control poly-A probe (Ventana Roche, Arizona, USA) was applied in parallel on all cases. A proven EBV-driven lymphoma was used as a positive control. Cases were defined as EBV-positive when the majority of viable tumor cells were EBER-positive.

Two antibody panels were applied to characterize the tumor (CD20, CD5, CD23, CD10, BCL-6, MUM-1, BCL2, Mib1, CyclinD1, TP53, MYC) and the viral protein expression pattern (LMP1, EBNA2, ZEBRA). All stainings were performed automatically according to manufacturers’ protocol.

Statistical analysis was performed using Statistica 7.0 (StatSoft) and consisted of univariate Kaplan-Meier analysis (log-rank and Wilcoxon test) and the Mann-Whitney non-parametric *t* test.

### Immunoglobulin (Ig) PCR and somatic hypermutation assay

*IGH* and *IGK* rearrangements of CLL and secondary DLBCL were studied by multiplex PCR with BIOMED-2 primers using a BIOMED-2 PCR based protocol [16]. The somatic hypermutation assay was performed on secondary DLBCL by standard methods from the clinical laboratory. The mutational status was ultimately determined using the IMGT database [17].

## RESULTS

### Case reports of EBV-positive Richter transformation

#### Case 1

In November 2000, a 72-year-old male patient presented with leukocytosis, compatible with a CLL, without lymphadenopathy. Cytogenetics showed del(11)(q22q24) resulting in loss of *ATM*. The patient received chlorambucil for 10 months. After 1 year, chlorambucil was restarted for increasing leukocytosis and lymphadenopathies. Because of disease progression in the bone marrow (91 % CLL cells; no transformation) and persistent thrombocytopenia, therapy was switched to fludarabine in April 2004. In December 2004 (after five administrations of fludarabine), the patient was admitted to the hospital because of general weakness, dizziness, and persistent cough. He had recently developed bilaterally enlarged cervical lymph nodes. EBV DNA had risen from undetectable levels (at time of diagnosis of CLL) to 16,100 copies/ml 2 weeks before biopsy and to 44,497 copies/ml at the time of biopsy. Histopathology revealed an EBER-positive non-germinal center B-cell DLBCL, with central necrosis. Cytogenetic analysis revealed evolution of the del(11q)-positive karyotype. The patient passed away shortly after one administration of rituximab, cyclophosphamide, doxorubicin, vincristine, and prednisolone (R-CHOP) due to the development of chemotherapy-induced pancytopenia complicated with neutropenic fever and sepsis.

#### Case 2

In October 2000, a 70-year-old male patient was diagnosed with CLL, but no treatment was installed. In May 2001, he developed an episode of CLL-associated idiopathic thrombocytopenic purpura, which responded well to steroids. In December 2002, chlorambucil was started because of increasing doubling time of leukocytes and development of cervical lymphadenopathies. In February 2003, fludarabine was initiated but stopped after two cycles because of persistent thrombocytopenia, and administration of steroids was restarted. In May 2003, the patient received four cycles of rituximab. Steroid administration could not be completely discontinued because of persistent thrombocytopenia. In January 2005, chlorambucil was restarted because of increasing lymphadenopathies. After four cycles, the patient developed a necrotizing suppurative right inguinal lymphadenopathy, without evidence for CLL localization. A CT scan showed atrophic changes and periventricular vascular leukomalacia in the brain. Retroperitoneal lymph nodes were significantly enlarged, with necrotizing center. A PET scan showed multiple moderately active mediastinal, mesogastric, retroperitoneal lymphadenopathies. Histopathology of a retroperitoneal mass revealed a CLL in transformation to a DLBCL, with central necrosis. Cytogenetics showed a complex karyotype including the del(13)(q14q22), characteristic for CLL. R-CHOP with a 50 % dose reduction of doxorubicin was started. Rituximab was stopped after one administration due to a severe hypotension. One week after CHOP, during the neutropenic phase, the patient passed away due to sepsis.

#### Case 3

In 2004, a 57-year-old female patient was diagnosed with CLL and treated with chlorambucil and fludarabine. She was lost to follow-up for 2 years during which she received four cycles of R-CHOP for which the indication is unclear. R-CHOP was discontinued and she remained in complete remission for 1.5 years. In 2009, she relapsed with CLL; cytogenetics of peripheral blood showed loss of 13q14 in the tumor cells. In December 2010, alemtuzumab therapy was started. This was accompanied by a gradual rise in the EBV DNA titer from undetectable levels to weakly positive. In April 2011, alemtuzumab was stopped because of severe pancytopenia. At that point, she was in partial remission: she still had some small lymphadenopathies in the inguinal and iliacal regions and at the liver hilus. In August 2011, the patient developed a thrombosis of the large saphenic vein due to compression by enlarged inguinal lymph nodes. Biopsy showed an EBV-positive DLBCL of non-germinal center B-cell origin clonally unrelated to the CLL. At that time, the EBV DNA titer peaked (1821 copies/ml). Following five cycles of rituximab monotherapy, the EBV DNA titer dropped to <500 copies/ml and the patient went in partial remission. Three and a half years later, she is in relatively good condition and she receives monthly substitution of IV immunoglobulins. Since follow-up after completion of rituximab therapy, the EBV titers gradually rose between December 2011 (3422 copies/ml) and July 2013 to 4135 copies/ml. Thus far, the patient is not developing new lymphadenopathies.

#### Case 4

In February 2000, a 62-year-old male patient with a history of prostate cancer (diagnosed in 1995) treated with prostatectomy, radiotherapy, and hormone therapy (Zoladex) was diagnosed with a common B-cell acute lymphoid leukemia (B-ALL). Cytogenetics revealed a t(1;19)(q23;p13) resulting in the E2A-PBX1 fusion. Complete remission was reached after two cycles of chemotherapy (vincristine, daunorubicin, methylprednisolone, and asparaginase) and three cycles of intrathecal methotrexate. In July 2011, further investigations were performed because of chronic fatigue and 4-kg weight loss over a period of 6 months. There was no morphological or molecular evidence for relapse of B-ALL (*E2A*-*PBX1*-negative by RT-qPCR). PET scan showed multiple faintly fluorodeoxyglucose-avid, slightly enlarged lymph nodes (bilateral axillar, retroperitoneal, iliacal, liver hilus, hepatogastric, mesenterial, retrocrural). A diagnosis of CLL was made and a watchful waiting approach was installed. All clinical and radiological parameters were stable for the next year.

**Fig. 1:**
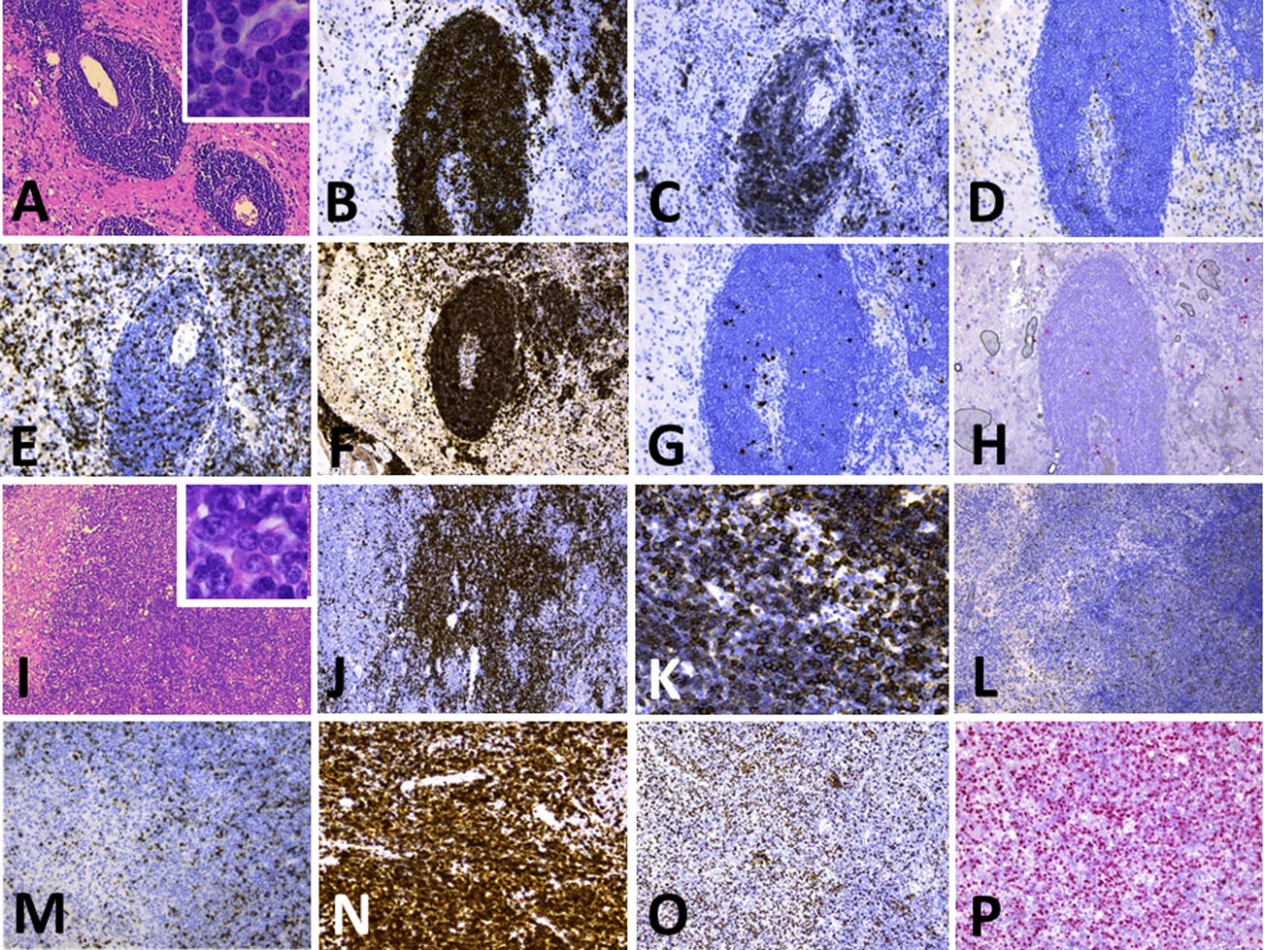
Histopathology of the brain biopsy of case 4 containing both CLL and Richter transformed CLL. (**a**)–(**h**) shows the presence of CLL in the brain; (**i**)–(**p**) shows the large cell Richter transformed diffuse large B-cell lymphoma (DLBCL) in the brain. **a**, **i** HE of perivascularly localized CLL (**a**) and the corresponding DLBCL (**i**). The *insets* show the tumor cells at larger magnification (×400). CLL and DLBCL were positive for PAX5 (**b**, **j**); however, CD20 was more weakly expressed in CLL (**c**) compared to DLBCL (**k**). Both components were negative for cyclin D1 (**d**, **l**). CD3 staining revealed the presence of scattered T cells (**e**, **m**); however, staining for the T-cell marker CD5 demonstrated strong expression in both CLL and DLBCL (**f**, **n**). Proliferation was limited in CLL (as evidenced by Mib1 staining, **g**) while the vast majority of DLBCL cells were actively proliferating (**g**). Notably, EBER ISH revealed some sparse positive small cells in CLL (**h**) and diffuse positivity in the large cells (**p**)

In October 2012, the patient was admitted to the hospital with sudden gait instability and two episodes of falling over the last 2 weeks. He complained of a tingling sensation in the right arm and leg. Over several months, he became progressively depressed and lost 4 kg. Clinical examination showed unstable gait, a tendency to fall to the left and a left central facialis paresis, but he had no headache, vertigo, loss of consciousness, fever, night sweats, or other associated symptoms. CT and MRI of the brain demonstrated a polylobular contrast-captating tumor in the right parietotemporal area with perilesional edema and mass effect, highly suspicious for a glioblastoma (Supplementary Fig. 1). Histopathological examination of the lesion, however, showed no evidence for a glioblastoma but revealed a dense lymphoid infiltrate with two components. The leptomeninges and superficial cortex showed a predominantly perivascular lymphocytic infiltrate, composed of small round lymphocytes with condensed chromatin and scant cytoplasm. Mitotic activity was virtually absent. The small cell component expressed the mature B-cell markers CD20, CD79a, and PAX5, and aberrant CD5 and CD23, but no CD10, CyclinD1, nor TdT. The deeper cortex showed a large blastoid lymphoid proliferation with extensive necrosis and increased mitotic and apoptotic activity. These cells had the same immunophenotype as the small-cell component, except for the presence of EBER in the large blastoid population and to a minor extent in the small cells. Laser microdissection was performed and DNA was extracted from the CLL and large-cell component. PCR confirmed the presence of a monoclonal B-cell population, identical to the CLL clone found in 2011, but different from the B-ALL clone in 2000. Diagnosis of a secondary central nervous system lymphoma with histological features of an EBV-induced RT of CLL to DLBCL was made (Fig. 1, Supplementary Table 2). The right parietotemporal lobe was irradiated (15 × 2 Gy), but the patient died 3 months later.

#### Case 5

In 1997, a 65-year-old male patient was diagnosed with CLL, with prognostically favorable (mutated *IgVH* gene status; CD38 negative, del(13)(q13q21)) and unfavorable del(17p) characteristics. From 2001 until 2003, he was treated with chlorambucil. In April 2006, chlorambucil was restarted because of recurrent lymphocytosis, mild anemia, and thrombocytopenia. Because of intolerance, chlorambucil was switched to fludarabine in June 2006. Because of persistent hepatosplenomegaly, fludarabine was discontinued after four cycles and alemtuzumab was started in October 2006. The patient received 12 cycles, but 2 weeks after the last injection, he presented with fever, diffuse abdominal pain, anorexia, but no B-symptoms. Bone marrow biopsy showed residual lymphoma without morphological evidence of transformation, but with cytogenetic evidence for clonal evolution. Inguinal and multiple retroperitoneal as well as mesenteric lymphadenopathies were present. Both spleen and liver were enlarged, and the latter showed multiple hyporeflective nodules at ultrasound. Liver function progressively decreased with concomitant abdominal ascites fluid accumulation. A liver biopsy revealed an EBV^+^ diffuse large B-cell lymphoma with post-germinal center phenotype. The patient was transferred to intensive care where his situation deteriorated. Two days later, the patient died due to disease progression.

#### Case 6

In 2003, a 58-year-old female patient with a history of lobular breast carcinoma was diagnosed with CLL. After 5 years of watchful waiting, she was included in the experimental arm of the Lucid trial (lumiliximab, fludarabine, cyclophosphamide, rituximab). After completion of the fourth cycle of rituximab in November 2009, she developed subungual bleedings and lymphadenopathies. After initial watchful waiting and rising EBV titers (from 9389 copies/ml in November 2009 to 42,671 copies/ml in February 2010), she developed B-symptoms. A bendamustine and rituximab regimen was initiated in March 2010 as an EBV-driven lymphoma was suspected. CT showed disease progression with multiple supra- and infradiaphragmatic lymphadenopathies and splenomegaly. Lymph node excision showed the presence of an EBV^+^ lymphoproliferation compatible with mixed cellularity type classical Hodgkin lymphoma (cHL), for which ABVD (doxorubicin, bleomycin, vinblastine, dacarbazine) was started. The lymphoproliferation was monoclonal, but the clonal relationship with the CLL was unclear due to incomplete Ig PCR results. Huge Reed-Sternberg cells showed JAK2 amplification, recurrent in cHL [18]. Due to disease progression, the patient died in November 2010.

### Summary of clinical and pathological results

The clinical and pathological characteristics of this series of 16 secondary B-cell lymphomas are summarized in Tables 1 and 2. CLL was diagnosed at a median age of 65 years (range 38–76 years). Aberrations characteristic for CLL (del 11q, 13q, 17p, trisomy 12) were found in all nine analyzed cases.

**Table 1:** Clinical features of 16 CLL cases with secondary B-cell lymphoma from UZ Leuven

Case	Sex	Age at Dx CLL (years)	Mutation status CLL	CLL therapy	Interval CLL/s BCL (age at sec BCL), years	Diagnosis	OS (months)	State	ROD
EBV^+^ secondary B-cell lymphoma									
1	M	72	ND	Chl, F	4.2 (76)	DLBCL	4.5	D	Sepsis
2	M	70	UNM	Chl, F	5.25 (75)	DLBCL	1	D	Sepsis
3	F	65	MUT	F, A	4.25 (69)	DLBCL	48	A	–
4	M	73	ND	None	1.3 (74)	DLBCL	4	D	Progression
5	M	65	MUT	Chl, F, A	9 (74)	DLBCL	1	D	Progression
6	F	58	MUT	Chl, FCR	7.25 (65)	cHL	8	D	Progression
EBV^−^ secondary B-cell lymphoma									
7	F	76	ND	None	0 (76)	DLBCL	11.75	A	–
8	F	72	ND	Chl	5.4 (77)	DLBCL	0.75	A	–
9	M	63	ND	FC, FCR	8 (71)	DLBCL	13.5	D	Progression
10	M	49	MUT	FC, CHOP	6.2 (55)	DLBCL	1.5	D	Progression
11	F	65	ND	F, FMD	1.6 (66)	DLBCL	10	D	Progression
12	M	38	UNM	FCR, A, alloBMT	3.9 (42)	DLBCL	9.5	A	–
13	F	57	UNM	Chl, F, O	8.8 (66)	DLBCL	3	D	Progression
14	M	54	UNM	F, A, alloBMT	2.2 (56)	DLBCL	4.5	D	Progression
15	M	65	ND	Chl, FCR	0.1 (65)	DLBCL	1.5	D	Progression
16	M	75	ND	Chl	2.9 (78)	DLBCL	32.5	D	CVA

*A* alive; *CLL* chronic lymphocytic leukemia; *cHL* classic Hodgkin lymphoma; *Chl* chlorambucil; *CVA* cerebrovascular accident; *D* dead; *Dx* diagnosis; *F* female; *F* fludarabine; *FC* fludarabine and cyclophosphamide; *FCA* fludarabine, cyclophosphamide, alemtuzumab; *FCR* fludarabine, cyclophosphamide, rituximab; *FMD* fludarabine, mitoxantrone, dexamethasone; *MUT* mutated; *M* male; *NA* not available; *ND* not determined; *O* ofatumumab, *sec BCL* secondary B-cell lymphoma; *UNM* unmutated; *Y* yes

**Table 2:** Pathological features of 16 CLL cases with secondary B-cell lymphoma from UZ Leuven

	CLL	Secondary B-cell lymphoma												
Case	Related aberrations	Related to CLL (by Ig PCR)	EBER/LMP1/EBNA2/ZEBRA	CD20	CD5	CD23	CD10	BCL6	MUM1	BCL2	Mib1	CyD1	TP53	MYC
EBV^+^ secondary B-cell lymphoma														
1	del(11q)	Y	+/+/+/−	+	+	Part	−	−	+	Weak	70 %	−	5 %	0 %
2	del(13q)	Y	+/NR/+/−	+	+	Part	−	−	+	+	90 %	−	5 %	5 %
3	del(13q)	N	+/+/−/−	+	+	−	−	+	+	+	75 %	−	50 %	10 %
4	ND	Y	+/+/−/+	+	+	+	−	−	+	−	80 %	−	5 %	10 %
5	del(13q), del(17p)del(13q) dem(13q)	NA	+/NA	+	NA	NA	−	Part	+	+	85 %	NA	5 %	NA
6	ND	NA	+/NA/NA/−	−	NR	−	−	−	+	+	NA	NA	90 %	10 % RS cells
EBV^−^ secondary B-cell lymphoma														
7	ND	Y	−	+	Weak	Part	−	+	+	+	80 %	−	5 %	10 %
8	ND	NA	−	+	−	−	−	−	NR	−	70 %	−	5 %	5 %
9	del(11q), del(13)	Y	−	+	+	Weak	−	−	−	−	80 %	−	75 %	1 %
10	del(17p)	Y	−	+	+	Part	−	−	−	+	80 %	−	80 %	1 %
11	ND	Y	−	Weak	+	+	−	−	+	+	90 %	−	1 %	5 %
12	13q	NA	−	+	+	+	−	Weak	+	+	30 %	−	30 %	1 %
13	ND	Y	−	+	+	Part	−	−	+	+	40 %	−	1 %	30 %
14	+12, del(17p)	Y	−	+	−	Part	−	−	+	+	80 %	−	60 %	25 %
15	ND	NA	−	+	+	Part	−	−	+	+	80 %	−	80 %	30 %
16	+12	Y	−	+	+	+	−	−	−	+	60 %	−	1 %	10 %

*CLL* chronic lymphoid leukemia, *EBER* Epstein-Barr virus-encoded RNA, *FISH* fluorescent in situ hybridization, *N* no, *NA* not available, *NR* not representative, *ND* not detected, *RS cells* Reed-Sternberg cells, *Y* yes

**Fig. 2:**
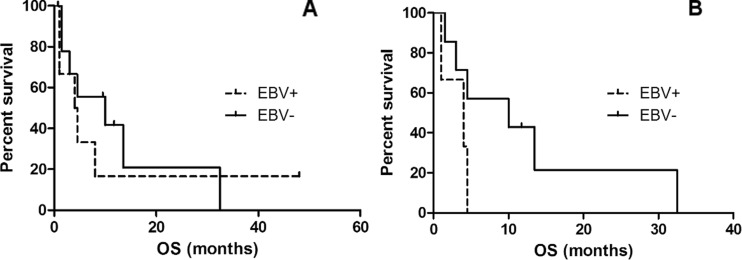
Survival analysis of secondary B-cell lymphoma. Kaplan-Meier plot comparing overall survival of all EBV-positive (*n* = 6, *dashed line*) and EBV-negative (*n* = 10, *full line*) secondary B-cell lymphoma (**a**, log-rank *p* and Wilcoxon *p* value >0.05) and of EBV-positive (*n* = 3, *dashed line*) and EBV-negative Richter transformation (*n* = 7, *full line*) (**b**, log-rank *p* and Wilcoxon *p* value >0.05) in CLL patients

Overall, 63 % of the secondary B-cell lymphomas occurred in male patients, at a median age of 70 years and following a median interval of 4 years after diagnosis of CLL. All were DLBCL of non-germinal center B-cell origin except for one case of classic Hodgkin lymphoma (no. 6). EBER was detected in 38 % (6/16) and was associated with higher MUM1 expression (100 % of EBV^+^ cases were positive compared to 67 % of EBV^−^ cases). Compared to the EBV^−^ cases, we observed a trend that EBV^+^ secondary lymphoma occurred later following CLL diagnosis (3.4 vs. 4.75 years, respectively, *p* value >0.05) and in older patients (median age 66 vs. 74 years, respectively, *p* value >0.05). There was no clear difference in prognosis (median survival 4.5 vs. 2.5 months, respectively, log-rank and Wilcoxon *p* value >0.05; Fig. 2a). The same observations were made when only the clonally related “true” RT cases were considered. Overall, three of four EBV^+^ and seven of seven EBV^−^ secondary lymphomas analyzed were clonally related to the CLL. Also in this series, there was a trend that EBV^+^ RT occurred later after CLL diagnosis (4.2 vs. 2.9 years, respectively, *p* value >0.05) in older patients (median age 75 vs. 66 years respectively, *p* value >0.05) and with a worse outcome (median 4 vs. 7.25 months, respectively, log-rank and Wilcoxon *p* value >0.05; Fig. 2b).

None of the comparisons was significant, likely due to small sample size.

Whereas the BCL2 protein was expressed in nearly all cases, high TP53 (50 vs. 33 %) and MYC (30 vs. 0 %) expression seemed more common in EBV^−^ than EBV^+^ RT (Table 2).

In the EBV^+^ cases, the EBER^+^ cells expressed an intermediate (LMP1+/EBNA2−, 2/4) or broad latency type (LMP1+/EBNA2+, 2/4). Notably, in one case (no. 4), the lytic protein ZEBRA was expressed in a small fraction of tumor cells indicative of active lytic replication.

## DISCUSSION

The Epstein-Barr virus (EBV) is an oncogenic human herpesvirus that has been associated with the development of lymphoproliferative disorders (LPD), in particular in immunocompromised individuals. It has been suggested that in some cases EBV may play a role in the transformation of indolent CLL to aggressive DLBCL, referred to as Richter transformation. According to current estimates, EBV is present in 6 % of the DLBCL variant of RT [19]. In our case series, however, EBV was present in 38 % (6/16) of DLBCL RT cases suggesting that the involvement of EBV in this disorder is underestimated. We therefore propose that newly diagnosed cases of RT are systematically screened for the presence of EBV by in situ hybridization, the most reliable method to determine the presence of EBV.

Interesting differences between the cases of with EBV^+^ secondary B-cell lymphoma were found.

Case 1 and 5 are classic cases of a fludarabine-associated EBV-driven LPD arising shortly after completion of therapy. Purine analogs, like fludarabine and cladribine, are highly effective treatments for low-grade B-cell neoplasms [20], but the accompanying long-lasting T-cell suppression, a major side effect, is related to the development of EBV^+^ LPDs [21, 22]. Less clear is the situation for case 2, who only received two doses of fludarabine and developed the LPD 34 months after. Intervals between completion of fludarabine and development of lymphoma were reported to be up to 1 year [21].

Case 3 developed a large B-cell lymphoma 2 months after completion of alemtuzumab monotherapy. Alemtuzumab targets CD52, present on nearly all B and T lymphocytes, monocytes, and natural killer (NK) cells, and is thus highly immunosuppressive. Prolonged T-cell deficiency induced by alemtuzumab is known to facilitate opportunistic infections and specifically to impair recovery of EBV-specific CD8+ T lymphocytes making the patient vulnerable for EBV-driven lymphoproliferation. Despite the large numbers of patients with chronic lymphocytic leukemia or T-prolymphocytic leukemia treated with alemtuzumab, the occurrence of EBV^+^ lymphoproliferative disease is very rare, especially during or following monotherapy [21, 23]. Whether there was a synergistic immunosuppressive effect with the fludarabine treatment this patient had received more than 4 years before is hard to define.

Case 4, with isolated RT of the brain, is unique for several reasons. Although asymptomatic secondary CNS infiltration by CLL has been shown at autopsy in 8 to 70 % of the cases [24–26], symptomatic involvement of the CNS by CLL cells is a relatively rare event. Isolated RT of the brain is even more rare: only 13 cases have been described in detail so far [11, 24, 27, 28], five of which had isolated leptomeningeal involvement, and eight demonstrated parenchymal involvement. Our patient had a history of different malignancies (prostate cancer, B-ALL, CLL, RT) but only received chemotherapy, including intrathecal methotrexate (MTX), for the B-ALL. MTX is frequently administered to patients with autoimmune diseases, especially rheumatoid arthritis, to suppress the hyper-immune state. Since the first case of lymphoma in a rheumatoid arthritis patient who received low-dose MTX was reported [20], MTX-induced immunosuppression has been linked to development of EBV-driven LPD [29]. However, the therapeutic or prophylactic administration of high-dose intrathecal MTX like in this case has—to our knowledge—not yet been associated with the development of EBV^+^ LPD of the brain.

As evidenced by cases, 3, and 6, sequential monitoring of EBV titers proved useful to predict the development of EBV-positive LPD in our patients, similarly to what has been described for post-transplant LPD patients [30]. We suggest that follow-up of EBV titers of CLL patients should be performed when evolution to RT is suspected, particularly in CLL patients receiving T-cell-suppressing therapy. This hypothesis is supported by a recent report suggesting that EBV reactivation may in fact be an under-diagnosed event in severely immunosuppressed CLL [31].

In contrast to DLBCL RT, the Hodgkin lymphoma variant of RT is rare and commonly positive for EBV. As for our case (no. 6), a retrospective study showed that mixed cellularity Hodgkin lymphoma is the most common subtype [32].

## CONCLUSION

Genetic studies have identified several lesions (abnormalities of *TP53*, *MYC*, *CDKN2A*, and *IGH*-mediated translocations) [11, 33, 34] associated with RT pathogenesis, and currently a number of genetic biomarkers (*NOTCH1* mutation, *IGHV4–39*) are used to identify CLL patients at risk of RT. Based on this small study, we propose EBV as an additional biomarker to predict RT development. Based on our observations, we suggest that EBV^+^ RT may be more frequent than is currently assumed. Larger series are required to validate our hypotheses.

## Electronic supplementary material

ESM 1 (DOC 100 kb).
