# Correction to: Secondary B-cell lymphoma associated with the Epstein-Barr virus in chronic lymphocytic leukemia patients

**DOI:** 10.1007/s12308-018-0323-5

**Published:** 2018-05-04

**Authors:** Julie Morscio, Emilie Bittoun, Nathalie Volders, Eveline Lurquin, Iwona Wlodarska, Olivier Gheysens, Peter Vandenberghe, Gregor Verhoef, Philippe Demaerel, Daan Dierickx, Xavier Sagaert, Ann Janssens, Thomas Tousseyn

**Affiliations:** 1 Department of Imaging and Pathology, Lab for Translational Cell and Tissue Research, KU Leuven, Leuven, Belgium; 2 Department of Pathology, University Hospitals Leuven, Leuven, Belgium; 3 Center of Human Genetics, University Hospitals Leuven, Leuven, Belgium; 4 Department of Nuclear Medicine, University Hospitals Leuven, Leuven, Belgium; 5 Hematology Department, University Hospitals Leuven, Leuven, Belgium; 6 Department of Radiology, University Hospitals Leuven, Leuven, Belgium

**Correction to: J Hematopathol (2016) 9:113–120**

10.1007/s12308-016-0273-8

The article Secondary B-cell lymphoma associated with the Epstein-Barr virus in chronic lymphocytic leukemia patients, written by Julie Morscio, Emilie Bittoun, Nathalie Volders, Eveline Lurquin, Iwona Wlodarska, Olivier Gheysens, Peter Vandenberghe, Gregor Verhoef, Philippe Demaerel, Daan Dierickx, Xavier Sagaert, Ann Janssens, Thomas Tousseyn, was originally published electronically on the publisher’s internet portal (currently SpringerLink) on 21 May 2016 without open access.

With the author(s)’ decision to opt for Open Choice the copyright of the article changed on 05 May 2018 to © The Author(s) 2018 and the article is forthwith distributed under the terms of the Creative Commons Attribution 4.0 International License (http://creativecommons.org/licenses/by/4.0/), which permits use, duplication, adaptation, distribution and reproduction in any medium or format, as long as you give appropriate credit to the original author(s) and the source, provide a link to the Creative Commons license and indicate if changes were made.

