# Supplementary material for: Secondary B-cell lymphoma associated with the Epstein-Barr virus in chronic lymphocytic leukemia patients
Source: J Hematop. 2016 May 21;9:113–20. doi: 10.1007/s12308-016-0273-8 (PMC5962620; doi:10.1007/s12308-016-0273-8)
Supplement: Supplementary file 1 — (DOC 100 kb) [file 12308_2016_273_MOESM1_ESM.doc]

**Supplement**

Supplementary Figure 1. Imaging of the brain in case 4

A: CT scan from 2011 demonstrates the absence of tumor lesions; B-C: T1 weighted coronal and sagittal MR image from 2012 showing a right temporoparietal contrast-captating mass with a necrotic center, mimicking glioblastoma (white arrow).


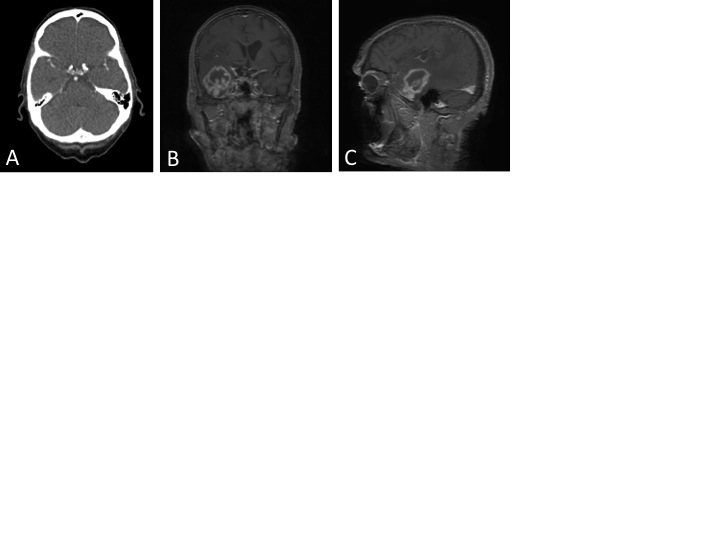


Supplementary Table 1

Ig PCR results from case 4 illustrating the clonal relationship between the RT and the B-CLL, but not with the B-ALL

|  | B-ALL | B-CLL | RT |
| --- | --- | --- | --- |
| IgH FR1-JH | M/337-353 | M/336 | M/336 |
| IgH FR2-JH | M/279-295 | M/273 | M/273 |
| IgH FR3-JH | M/139-154 | M/136 | M/136 |
| IgK VK-JK | P | M/199 | M/149-199 |
| IgK VK-Kde | MP/208 | N | M/233 |
| TCRB VB-JB1/2 | MP/259 | P | MP/255-265 |
| TCRG VG-JG | M/182 | P | P |
